# Satisfaction with use of public health and peer-led facilities for HIV prevention services by key populations in Nigeria

**DOI:** 10.1186/s12913-019-4691-z

**Published:** 2019-11-21

**Authors:** Bartholomew Ochonye, Morenike Oluwatoyin Folayan, Adesegun O. Fatusi, Godwin Emmanuel, Oluwatomi Adepoju, Babatunde Ajidagba, Toluwanimi Jaiyebo, Paul Umoh, Ayo Yusuf

**Affiliations:** 1 Heartland Alliance International, Abuja, Nigeria; 2 New HIV Vaccine and Microbicide Advocacy Society, Lagos, Nigeria; 3 Department of Child Dental Health, Obafemi Awolowo University, Ile-Ife, Nigeria; 4 College Research and Partnership Advancement (CoRPA) Office, College of Health Sciences, Obafemi Awolowo University, Ile-Ife, Nigeria; 5 Academy for Health Development (AHEAD), Ile-Ife, Nigeria

**Keywords:** Key populations, Nigeria, Stigma, Public-health facilities, Peer-led organisations, Same-sex prohibition law, Health services, HIV prevention services

## Abstract

**Background:**

The aim of the study was to identify the proportion of female sex workers, men who have sex with men, and people who inject drugs who had accessed HIV prevention services at public health facilities and peer-led facilities, their level of satisfaction with these services, and perceived barriers and challenges to accessing HIV services from public and peer-led HIV prevention service providers.

**Methods:**

A mixed-method approach was used to collect data from key populations in the four states in Nigeria. Quantitative data collected included level of satisfaction with and barriers to use of public and peer-led facilities. In-depth interviews and focus-group discussions were conducted to explore reasons for satisfaction with and barriers to use of services. Descriptive and bivariate analyses were was conducted for quantitative data. Qualitative data were summaried, emerging themes identified, described and quotes reflecting the themes corresponding to interview questions highlighted.

**Results:**

Nine hundred sixty-seven persons responded to questions on the use of public health or/and peer-led facilities. Two hundred thirty-eight (49.4%) respondents had received HIV and sexual and reproductive health services through public health facilities, and 236 (48.7%) had received the services through peer-led facilities. Significantly more respondents were satisfied with the quality of services provided by peer-led organisations than with public health facilities with respect to service providers listening to respondent’s problems and concerns (*p* = 0.007),privacy and confidentiality (*p* = 0.04) and respect of rights of service recipients (*p* = 0.04). Significantly more respondents using peer-led organisations than those using public health facilities identified no barriers to service access (*p* = 0.003). More respondents using public health facilities than peer-led facilities identified cost of services (*p* = 0.01), confidentiality (*p* = 0.002), waiting time (*p* < 0.01) and staff attitude (*p* = 0.001) as barriers to service access. Thee was no difference in the proportion of respondents willing to discontinue their use of either facilities (*p* = 0.08). Qualitative data revealed that concerns with access of services at the public health facility were due mainly to stigma and the effects of the same-sex prohibition law.

**Conclusion:**

Key populations were more satisfied receiving HIV prevention services at peer-led organisations than at public health facilities.

## Background

Despite their greater vulnerability to HIV, key populations at high risk of HIV exposure have been inadequately served by national HIV and AIDS responses in most African nations [1]. The same is true of the Nigerian HIV response. There is, however, willingness to scale up current efforts on HIV programming for female sex workers (FSW), men who have sex with men (MSM), and people who inject drugs (PWID) in Nigeria. One of the efforts at appropriate programming appropriately for these key populations has been estimation of the size of the population and identification of ‘hot spots’ [2]. The development of national guidelines for HIV prevention programming for FSW [3] and PWID [4] has helped in the design and implementation of comeparable programmes, using a combination of HIV prevention strategies.

Despite these efforts, there are gaps in the HIV prevention programmes for key populations. The estimate of key populations in Nigeria is believed to be an underestimation [5, 6], resulting in negative impact on resource allocation for the community. At the time this study was conceived, in 2013, there were no HIV prevention service programmes led by FSW and PWID, and just five community-led MSM groups offered programmes to peers. The networks of key populations have limited capacity to advocate for their community needs. Interventions to promote behaviour change were adapted from approaches primarily designed for heterosexuals in the general population. In contrast, emerging promising initiatives use a rights-based approach for HIV programming for MSM, and there is a commitment to community empowerment of FSW by the national government, using the cluster models of service delivery [3].

However, restrictive policies, legal and societal environments are formidable barriers for programming with PWID, FSW and MSM [7]. These factors are exacerbated by the poor health systems infrastructure [8]. Also, the risks of arrests [1], oppression [9] and imprisonment [10] of FSW, MSM, and PWID were not considered when the national programmes for each key population were designed. There are few programmes to improve access to justice for victims of violations, and the national prevention and HIV treatment guidelines [11] do not address lack of access to services for those in detention or in prison. There is clearly a need to develop systems and structures that can deliver effective HIV prevention, treatment, care and support for key populations in Nigeria.

The peer-led HIV service provision programmes initiated in Nigeria by the Population Council and Heartland Alliance had shown that peer-led programmes that target key populations have high potential for success and impact [12]. However, peer-led programmes for prevention, treatment, care and support are few and may be inadequate to meet the needs of the population. Thus, public health services are still required to provide health care services for key populations in Nigeria.

Access to services through the public health care system in Nigeria is challenging to many persons. The challenges include travel distance [13], cost of services [14], unfriendly service hours [15], delay in service provision [16], and unfriendly and stigmatising health care providers [17]. Unfriendly and stigmatising behaviour of health care providers have been especially troublesome for key populations [15].

This study assessed the proportion of FSW, MSM and PWID who had accessed HIV prevention services from the public health facilities and peer-led initiatives, their level of satisfaction with these services, and perceived barriers and challenges to accessing HIV services from public and peer-led HIV prevention service providers. The findings will provide evidence that can be used to improve access to HIV prevention service for key populations in Nigeria.

## Methods

### Study design, study site and study populations

Data were extracted from the clients’ service utilisation component of a larger data set that had been generated to provide baseline information on FSW, MSM and PWID for programmatic purposes**.** Data were generated by use of a sequential mix-method approach, wherein findings from the quantitative data were explored further by using qualitative data.

Data were collected from a sample of FSW, MSM and PWID residing in four of the 36 states in Nigeria: Enugu, Nassarawa, Benue, and Akwa-Ibom. The study sites were limited to these states because the data were to be used to plan and implement HIV prevention programmes for the target populations in them as part of a targeted intervention for key populations funded by the Global Fund for AIDS, Tuberculosis and Malaria. The intervention in the four states was named the Enhancing Key Population Intervention in Nigeria through Capacity Development (EKPIN) project. The study adopted the client-centred access to health care framework developed by Levesque and colleagues [18]. Experience of care dimensions of the World Health Organisations quality of care framework for maternal and newborn health care was integrated into the theoretical framework for the design of this study so it can address the quality of care issues [19].

All study participants were 18 years of age or older and provided written consent for participation. The extracted data consisted of quantitative and qualitative data on clients’ satisfaction with services offered by the public health sector and the peer-led service organisations.

### Quality assurance

Field workers were selected on the basis of their competence and experience. The workers were also trained before data collection commenced and were rigorously supervised during data collection. During the project, all field workers uploaded the quantitative data and transmitted audio-recordings of the interviews daily. The study coordinator reviewed documents and sought clarification and rectification promptly. The study co-PI also reviewed samples of the data.

### Ethical considerations

The Institute of Public Health Institutional Review Board gave ethical clearance for the conduct of the study (IPHOAU/12/268). Study participants were duly informed about the objectives of the study, risk and benefits, voluntary nature of study participation, and freedom to withdraw from the study at any time. Written consent was obtained from all participants. No participant identifiers were collected. The research outcomes were shared with 61 representatives of the MSW, FSW and PWID communities and with policy makers and HIV programmers for validation of results.

Data were collected by community members in locations that were considered safe and private. In all the states, a peer-led non-governmental organisation setting was used for data collection except when the respondent preferred an alternative spaces. Privacy and confidentiality of participants was ensured throughout the study. All study participants signed an informed consent form, were provided with educational material on prevention and treatment of HIV, received ₦2, 000 ($11.50) for transportation reimbursement, and were given condoms and lubricants.

### Quantitative study

This aspect of the study sought to identify the proportion of FSW, MSM and PWID who had accessed HIV prevention services from the public health facilities and peer-led initiatives and assess their their level of satisfaction with these services.

#### Sample size

A standard sample-size calculation approach for cross-sectional study was used to determine the sample size for the study using WINPEPI software [20]; a minimum sample of 500 study participants was required (125 participants per state). A minimum of 50 FSW, MSM and PWID were to be recruited from each state.

#### Participants’ recruitment and data collection

Study participants were recruited between April and June 2015 from a minimum of two different parts of each state that had been mapped to have clusters of MSM, FSW and PWID. Decisions on the site for study participants was made based on the report of the key populations estimated size [5]. The target was 25 FSW, 25 MSM and 25 PWID recruited from each of the two sites in each of the four states.

Interviewers administered a semi-structured questionnaire developed in English, to study participants. The study instruments were questions that were adapted from the instruments used in the National AIDS and Reproductive Health Survey. Key words and phrases, especially sensitive ones, were translated to the languages of each selected community and collected in a list generated during the training of interviewers. Interviewers used this material as a reference when in the field. A similar technique had been used for past national reproductive health survey [21–25] and the study by Folayan et al. [26].

First, study participants were asked if they had used public or peer-led facilities to access HIV prevention services. Those who had used services where then asked about their level of satisfaction with the services, using a 5-point likert-like scale: 1, very dissatisfied; 2, satisfied; 3, neutral; 4, satisfied; and 5, very satisfied. For the purpose of analysis, the categories ‘satisfied’ and ‘very satisfied’ were were collapsed into ‘satisfied, and ‘dissatisfied’ and ‘very dissatisfied’ were collapsesd into ‘dissatisfied.’ Respondents stated how satisfied they were for three service quality indicators: a) extent to which service providers listened to their problems and concerns; b) extent to which service providers assured them of confidentiality and privacy; and c) extent to which service providers respected their rights as a service recipient.

Next, respondents who had accessed HIV prevention services in public or peer-led organisations were asked to identify barriers to accessing these services: distance to services, cost, confidentiality, waiting time, hours of operations, and staff attitude. Respondents also had the option of identifying if they had perceived no barrier and to list barriers to accessing services beyond those specified in the questionnaire. Finally, respondents were asked about their willingness to continue to seek HIV prevention care in the public or peer-led facilities, or if they would rather seek services elsewhere.

#### Data collection procedure

Four field workers and focal persons for MSM, FSW and PWID received a three-day training on the study objectives; methodology, including ethical approach to data collection; and community-entry procedures. Field workers had prior experience conducting national reproductive health surveys and understood the local language and target community in each site. The field workers and focal persons pilot-tested the instrument and made recommendations for revisions before its printing. For each site, a local community guide was designated to facilitate community mobilisation and community entry processes. The guides were instructed in the study objectives and methodology; in making initial contacts with non-government organizations that were working with key populations in the state; in facilitating recruitment (locating key populations’ hot spots); and in booking study-related appointments. A one-day training was conducted for the local community guides. The study in each state was overseen by an experienced researcher who served as the state supervisor.

Study participants were recruited through exponential non-discriminatory snowball sampling [27]. “Seeds” were recruited from members of the target groups, who then recruit other members from their personal networks. “Seeds” refer to the first contacts made for the snowballing; they were identified by state contacts engaged with planning of the study. Initially, three seeds were recruited, one for each key population. After filling the questionnaire, (s)he was asked to recruit a maximum of three more peers into the study. This recruitment procedure was expected to yield a wide spectrum of community members engaged in the study.

#### Data analyses

Univariate analysis was carried out to determine the proportion of respondents who had received HIV and sexual and reproductive health services from public sector or a peer-led service and their level of satisfaction and perspectives on barrier to services. Bivariate analysis was used to compare findings between the key population groups by use of chi-square test and Fishers exact test where appropriate. Analyses were conducted with SPSS. Statistical significance was specified at a *p* value less than 0.05.

### Qualitative study

This aspect of the study further explored key populations’ perceived barriers to accessing HIV services from public or peer-led HIV services. Forty in-depth interviews (10 per state) were conducted with leaders of key population communities and 16 (4 per state) focus group discussions with key populations. The sessions were audio-recorded, and hand-written notes were taken. Basic socio-demographic data were collected for the participants, and the research personnel developed a detailed debriefing note and a summary report immediately after each interview or discussion session.

#### Participants’ recruitment and data collection

Study participants recruited for the in-depth-interviews were community leaders who are highly knowledgeable on issues of key populations’ access to HIV prevention, treatment and care services. The community leaders in each state were identified through the national key populations secretariat. The leaders are persons who represent the communities in key strategic national and state policy and program meetings, and whom the communities recognise as their political representatives. The interviews solicited the participants’ perspectives about structural and other barriers to key populations’ access to HIV prevention service. The in-depth interviews were conducted with a semi-structured guide that was based on the study objectives, extant literature, and input from representatives of key populations.

With respect to the focus-group discussions, one individual session for each population (FSW, MSM and PWID) was held in each state, and one combined session of the groups was also held. Each session had 6–10 participants. The sessions started with an “ice breaker,” a short briefing on the EKPIN project, and an opportunity for participants to ask questions about the project and the research. A vignette was used to facilitate the discussions by allowing participants to project their views rather than personalise the discussion. For each session, the discussants were guided to develop the profile and identity of the individual to be discussed in the vignette. Thus, the names and sociodemographic profile of the individuals used for each vignette were appropriate for the focal key population group and the study location. The issues discussed were the same, however, in all the focus group discussions,

#### Data analyses

The qualitative data generated from the in-depth interviews and focus-group discussion included handwritten notes (brief field notes, summary notes and debriefing reports) and transcripts from audio recordings. In-depth analysis was conducted using the Atlas.ti software package. Three designated team members worked in collaboration with two expert qualitative data analysts to develop a codebook and guidelines suitable for use with Atlas.ti. A selected set of texts was double-coded by two analysts to establish intra-coder and inter-coder reliability – a quality control measure that was automatically generated by Atlas.ti. After this quality check was completed, the coding team discussed the coding discrepancies and resolved them by consensus during several face-to-face sessions. The process continued until inter-coder reliability was at least 80%. The remaining text then was coded by one analyst, with regular discussions among all the analysts to achieve standardisation and reliability. Themes for each of the questions were identified and a streamlined list of quotes developed.

## Results

### HIV service utilisation and users’ rating of service quality

Of the 500 recruited respondents, 482 (96.4%) responded to questions on the use of public health services and 485 (97.0%) on the use of peer-led services (Table 1). About an equal percentage had received HIV and sexual and reproductive health services from public health facilities (49.4%) and from peer-led organisations (48.7%). Almost half of the respondents indicated that they had used both types of service. FSW recorded the highest proportion (57.4%) that had received services from the public sector, followed by MSM (47.5%). MSM recorded the highest proportion (53.5%) that had received services through peer-led organisations, followed by PWID (51.0%). Statistically significant difference was recorded among the groups in the use of public sector health facilities (*p* = 0.01) but not with use of services through peer-led organisations (*p* = 0.13). A significantly higher proportion of FSW accessed HIV services through the public health sector than through peer-led organisations (57.4% vs 43.0%; *p* = 0.005), but this was not the case for MSM (47.5% vs 53.5%; *p* = 0.32) or PWID (41.2% vs 51.0%; *p* = 0.09) (Not shown in Table).

**Table 1:** Percentage distribution of female sex workers, men who have sex with men and people who inject drugs who access services at public (government) health institutions and peer-led organisations

	Public health institutions				Peer – led organisations			
	Total number of respondents	Number who had received services	Proportion of total %	*p* value	Total number of respondents	Number who had received services	Proportion of total %	*p* value
Female sex workers	188	108	57.4	0.01	186	80	43.0	0.13
Men who have sex with men	141	67	47.5		144	77	53.5	
Persons who inject drugs	153	63	41.2		155	79	51.0	
TOTAL	482	238	49.4		485	236	48.7	

As illustrated in Table 2, the proportion of respondents that expressed satisfaction with the three service quality indicators in the public health facility ranged from 74.6 to 76.4%, whereas the proportion of respondents that expressed satisfaction with the indicators for peer-led organisations ranged from 90.4 to 90.8%. These differences in respondents’ satisfaction between the two sources of service were statistically significant with respect to service providers listening to respondent’s problems and concerns (*p* = 0.007), privacy and confidentiality (*p* = 0.04) and respect of rights of service recipients (*p* = 0.04).

**Table 2:** Percentage distribution of respondents by level of satisfaction with HIV-related services offered by public (government) health institutions vs peer-led organisations

Service quality indicators	Public sector health facilities				Peer-led organisations				Difference in proportion of persons satisfied with service quality: public vs peer-led organisation services (*p* value)
	Dissatisfied n (%)	Neutral n (%)	Satisfied n (%)	Total	Dissatisfied n (%)	Neutral n (%)	Satisfied n (%)	Total	
**a.** Satisfaction with the extent to which service providers listened to respondent’s problems and concerns	28 (12.0)	27 (11.6)	178 (76.4)	233	9 (3.9)	12 (5.3)	206 (90.7)	227	0.007
**b.** Satisfaction with the extent to which service providers assured respondent of confidentiality and privacy	33 (14.0)	27 (11.4)	176 (74.6)	236	10 (4.3)	11 (4.8)	208 (90.8)	229	0.04
**c.** Satisfaction with the extent to which service providers respected respondents’ rights as a service recipient	33 (14.0)	27 (11.4)	176 (74.6)	236	9 (3.9)	13 (5.7)	207 (90.4)	229	0.04

As illustrated in Table 3, most service users were willing to use the same facilities again, and there was no significant difference in willingness between recipients of care in the two kinds of facility (public health facilities, 65.2%; peer-led facilities, 69.1%%. *P* = 0.38).

**Table 3:** Percentage distribution of respondents who had previously used public and peer-led HIV to receive SRH services by their desire to continue to use the same health facility

Variables	Public health institution				Peer-led institution			
	will seek another health facility	will use same facility	not sure	Total	will seek another health facility	will use same facility	not sure	Total
	n (%)	n (%)	n (%)	N (%)	n (%)	n (%)	n (%)	N (%)
Female sex workers	23 (23.0)	72 (72.0)	5 (5.0)	100 (100.0)	17 (23.3)	52 (71.2)	4 (5.5)	73 (100.0)
Men who have sex with men	20 (30.8)	43 (66.2)	2 (3.1)	65 (100.0)	15 (21.4)	50 (71.4)	5 (7.1)	70 (100.0)
Persons who inject drugs	27 (43.5)	33 (53.2)	2 (3.2)	62 (100.0)	19 (25.7)	48 (64.9)	7 (9.5)	74 (100.0)
TOTAL	70 (30.8)	148 (65.2)	9 (4.0)	227 (100.0)	51 (23.5)	150 (69.1)	16 (7.4)	217 (100.0)

### Perceived barriers to HIV Services for key Populations

Table 4 illustrates the proportion of respondents that identified barriers to access of services. Of the 238 respondents who had received services in public health facilities, 134 (56.3%) identified no barriers to service access, which was significantly less than those who had received services in peer-led facilities (164 (69.5%) vs 134 (56.3%); *p* = 0.003). More key populations identified cost of services (*p* = 0.01), confidentiality (*p* = 0.002), waiting time (*p* < 0.001), staff attitude (*p* = 0.001) as a barrier to accessing services in public health facilities than in peer-led organisations.

**Table 4:** Percentage distribution of respondents by barriers encountered in accessing the services provided by public health facilities or peer-led organisations

Variables	Public health facilities					Peer-led organisations					Difference between public and peer facilities (*p* value)
	FSW *N* = 108 n (%)	MSM *N* = 67 n (%)	PWID *N* = 63 n (%)	Total *N* = 238 n (%)	Key population group comparison (*p*-value)	FSW *N* = 80 n (%)	MSM *N* = 77 n (%)	PWID *N* = 79 n (%)	Total *N* = 236 n (%)	Key population group comparison (*p*-value)	
Distance to service	12 (11.1)	11 (16.4)	12 (19.0)	35 (14.7)	0.31	4 (5.0)	9 (11.7)	16 (20.3)	29 (12.3)	0.01	0.44
Cost of services	7 (6.5)	5 (7.5)	9 (14.3)	21 (8.8)	0.28	0 (0.0)	1 (1.3)	7 (8.9)	8 (3.4)	0.007	0.01
Confidentiality	6 (5.6)	6 (9.0)	12 (19.0)	24 (10.1)	0.03	2 (2.5)	3 (3.9)	2 (2.5)	7 (2.9)	0.79	0.002
Waiting time	13 (12.0)	8 (11.9)	15 (23.8)	38 (16.0)	0.12	1 (1.25)	3 (3.9)	7 (8.9)	11 (4.7)	0.11	< 0.01
Hours of operation	3 (2.8)	6 (9.0)	7 (11.1)	16 (6.7)	0.07	4 (5.0)	2 (2.6)	5 (6.3)	11 (4.7)	0.57	0.33
Staff attitude	11 (10.2)	8 (12.0)	14 (22.2)	33 (13.9)	0.11	3 (3.8)	4 (5.2)	4 (5.1)	11 (4.7)	0.94	0.001
Others	3 (2.8)	0 (0.0)	2 (3.2)	5 (2.1)	0.40	0 (0.0)	2 (2.6)	0 (0.0)	2 (0.8)	0.11	0.26
No barrier	62 (57.4)	40 (59.7)	32 (50.8)	134 (56.3)	0.80	58 (72.5)	54 (70.1)	52 (65.8)	164 (69.5)	0.40	0.003

*FSW* female sex workers, *MSM* men who have sex with men, *PWID* people who inject drugs

Significantly more PWID identified confidentiality (*p* = 0.03) as a barrier to accessing services in public health facilities. Also, significantly more PWID identified distance to services (*p* = 0.01) and confidentiality (*p* = 007) as a barrier to accessing services in peer-led organisations..

Findings from the qualitative study provide further insights into perceived barriers to accessing services. Respondents especially identified stigmatisation as a barrier to using public sector facilities – stigmatization from the health care workers; fear of being recognized and ‘labelled;’ and stigmatisation by family members, friends, and community members who may learn of their status from sources within the public health facilities, as the services are used by every group. Examples of reponses are these:

*“If you go to hospitals for check-up, there are some kinds of behaviours that the health personnel show to MSM community members. Most especially if you have something in your anus like genital warts which the nurses are expecting to see on your penis or in your vagina, and they see it in your anus; they will just lay a stigma or hatred on you*.*……..****MSM, IDI, Nasarawa***

*I want to be very realistic. In Nigeria we have a law against gays. Tayo (i,e the character portrayed in the vignette for the FGD in the specific setting) will be scared of accessing public health facilities because he’s afraid of 14 years jail term. ……* ***MSM, FGD, Akwa-Ibom.***

*Yes, Tayo cannot access any public health facility because he might feel that he is going to be stigmatized. So it is proper for him or it will be wise if a healthcare facility for key populations is being established for Tayo to be able to access his healthcare, in order not to be stigmatized.……..****MSM, FGD, Akwa-Ibom***

Access of HIV prevention services given through peer-led organisations was adjudged to be more effective and was preferred over care given through public health facilities. Respondents indicated that they felt more comfortable sharing problems encountered in their daily work and lives with peers manning peer-led facilities, whereas they had feelings of shame, guilt and embarrassment when discussing these issues with healthcare workers in public sector settings.

*“She (i.e. the key population character in the vignette) will feel good because it is young people that will take care of her and that one is better because they will be of almost the same age and they will advise her.”* ***…….FSW, FGD, Nasarawa***

*“He (i.e. the key population character in the vignette, , in this case a PWID) will like to come to an organisation that is run by drug users because he believes that sharing himself with a drug user, everything is confidential and whatever he is doing there remains there and die there.* ***.....….PWID, FGD, Enugu.***

## Discussion

Key populations play an important role in HIV epidemics nationally and globally, as they contribute disproportionately to new HIV infection. Unfortunately, research on key populations has been sparse, and little attention has been given to their access to services and the quality of services they receive. This study addressed this critical research gap and, to the best of our knowledge, is the first to objectively compare the use of HIV prevention services by key populations through public health facilities and peer-led organisations in Nigeria.

The study revealed key findings regarding service utilisation and satisfaction with services. First, about equal portions of the key populations accessed HIV prevention services from public health facilities or peer-led organisations. Second, while more FSW access services through public health facilities than peer-led facilities, there was no significant difference in the proportion of MSM and FSW that access both services. Third, most service users were satisfied with the services they received from both public health facilities and peer-led facilities and were willing to use the same services again. The proportion of service users that expressed satisfaction with the services received was, however, significantly higher for peer-led organisation than for public sector health facilities. Fourth, respondents face barriers to accessing HIV prevention services, with sources of barriers higher in public health-sector facilities than in peer-led services. A major source of barrier was stigma and the effects of the same-sex prohibition law. Also, the health care experiences of key populations are not monolithic with the level and type of barriers faced varying among the groups.

Access of key populations to HIV prevention services is critical in the fight against HIV, as these populations constitute a large proportion of the HIV burden -- 54% of all new HIV infections in 2018 [28]. Key populations are disproportionately affected by HIV nationally and globally, with higher risks of acquiring HIV than in the general population (age 15–49): 21 times higher for FSW, 22 times higher for MSM, and 22 times higher for PWID [28]. The increased risk is influenced not only by risky behaviour among the groups, as documented in Nigeria [29–31] and elsewhere [32, 33], but also by inadequate availability of HIV-related services and by social and legal constraints on access of key populations to the services. In Nigeria, the same-sex marriage prohibition act inhibits access of HIV related services by the key populations [34], and it may be responsible for the low proportion of our study respondents that accessed HIV prevention services, either through public health facilities or peer-led organisations.

Our findings on barriers to accessing services identified lack of assurance of confidentiality as a significant barrier to accessing public health sector facilities. Other significant barriers were cost of services, waiting time, and staff attitude. The proportion of the key populations that had accessed HIV prevention services was low, but the proportion falls within the range of that for the use of other facility-related care in Nigeria; for example, only 43% of pregnant women delivered in a health facility in 2018 [35]. Thus, the health behaviour of the key populations appears to follow that of the general pattern of the Nigerian population. This troubling finding implies that more than half of the key population have not accessed HIV prevention service from either public sector health facilities or peer-led service organisations. This figure falls significantly below the national HIV response target of 90% [36]. Integrating HIV services into the mainstream of health services is a strategy for improving the access of key populations to HIV prevention, treatment and care services [37, 38]. Without correction of the stigmatising behaviour of health workers and other service-related barriers, utilisation of the services will continue to be sub-optimal, even if the services are made available. A systematic review of determinants of access to HIV testing services by FSWs in sub-Saharan Africa, for example, identified approachability, acceptability, availability, affordability and appropriateness of the services as crucial factors [39].

Though our findings revealed a higher preference for peer-led services than for public health sector services, the proportion of key population members that accessed HIV prevention services from peer-led services was not higher than that of public sector facilities. This difference may be because peer-led services are few in Nigeria and are far apart [40, 41], making distance to services a significant limiting factor for service access, as highlighted in this study. The higher preference for peer-led services is intuitively expected, as they are likely to give a higher level of connectedness and empathy.

Our findings highlight the need to increase the availability of peer-led services and the support that will keep them optimally functional, as they can bridge the shortfall between the supply and demand for services in the public sector health facilities. Services provided by peer-led organisations are donor funded and not linked with public health facilities in Nigeria. Programmes are funded by organizations such as the United States Agency for International Development [42], Centre for Disease Control [43], Global Fund [44], and United Nations Office on Drugs and Crime [45], with no nationally or state funded HIV prevention programme for key populations in Nigeria. The unfriendly political and legal environment in Nigeria, resulting from punitive laws against FSW, MSM and PWID [46, 47], regrettably constrains national and state government investment in HIV prevention programming for key populations. For a country where key populations contribute significantly to the HIV incidence and prevalence, and where peer-led services can significantly augment the national HIV response for key populations, the lack of government investment in key population response seriously diminishes HIV control.

The level of satisfaction with services and the high proportion of service users who indicated willingness to continue using the services (either public sector health facilities or peer-led services) is striking. This finding contrasts with that reported for key populations in Bangladesh, where most service users indicated unwillingness to return to public health facilities because of perceived low quality of care [48]. On the one hand, our result may imply that the quality of HIV prevention services in the focal Nigerian states is good and acceptable, which is in contrast with most reports about the quality of health care in the country [49–52]. On the other hand, the responses may reflect respondents’ high tolerance for poor-quality health services, or resignation that facilities where they accesses care are the only ones within reasonable travel distance.

This study has limitations. These includes reliance on the respondents’ statements, with little opportunity to check their validity and reliability. Also, the study is cross-sectional, so no causality can be adduced to the relationship between any action and outcomes. Our findings on the barriers to access of health care services by key populations may also have been limited in comprehensiveness. Nevertheless, the study is important for a number of reasons. First, it addressed a research gap in reports to date. Second, it illustrates that concerns about abuse of human rights are not unique to public health facilities. Third, the findings have implications for programming HIV prevention services for key populations. Public health facilities and peer-led organisations serve the needs of key populations. It is therefore important that barriers to accessing services in public health facilities be addressed to encourage key populations to use the available HIV prevention services. Regular training in ethics, confidentiality and medico-legal issues could help improve health care delivery in both public and peer-led organisations. On-the-job training of health care providers could improve their listening skills and assurance of client confidentiality. Hospital waiting times could be shortened. These improvements could increase the uptake of services by key populations as well as the general public, and they could help in preparing for future integration of peer-led facilities into government-funded systems.

## Conclusion

This study found that the proportion of FSW, MSM and PWID that had accessed HIV prevention services from either public health facilities or peer-led organisations in four states in Nigeria was low. Nonetheless, a high proportion of those who received services from either sources were satisfied with the services provided and would continue to seek service in the same facilities. However, a higher proportion of respondents reported facing barriers to accessing services at public health facilities than at peer-led facilities. Investments are needed to increase the number of key populations that access HIV prevention services in Nigeria, a country where HIV control will not be possible without committed and successful prevention programmes for high-risk populations.

## Data Availability

Study related data and materials are accessible on request from the corresponding author.

## Abbreviations

AIDS: Acquired Immunodeficiency Syndrome
FSW: Female Sex Worker
HIV: Human Immunodeficiency Virus
MSM: Men who have Sex with Men
PWID: People who Inject Drugs
